# A Case of Temporary Mental Alienation

**Published:** 1844-12

**Authors:** 


### SELECTIONS

FROM

### AMERICAN AND FOREIGN JOURNALS.

---

*A Case of Temporary Mental Alienation.*—About noon, on the 7th of May last, I was called to see M. J., a young man belonging to a distinguished family, and residing in the *Rue de la Paix.* His temperament is sanguine, his disposition amiable. I found him upon his bed, where it was with the utmost difficulty that four robust men could hold him. He recognized no one, not even his friends or relatives. His face was highly flushed, his eyes wild, haggard, and rolling in their orbits. He had a violent delirium, characterized by a fixed and false idea. He saw, before him, the corpse of a man, and gazed at it with sneers. He wished to drink the blood of the imaginary being, and demanded a cup of it filled high unto the brim. Hereupon his tongue was moistened with a few drops of water, and he believed himself to be drinking blood. He asked for more, and swallowed a tumbler full with avidity. He now bade his friends farewell, expressing a determination to end his torments by self-destruction. He should die happy, since he was revenged upon the man who haunted his vision. But immediately he reproached himself for his atrocity, and expressed his shame and condemnation for the infamous act that he had committed. He was seized with nausea, (probably from the disgust of drinking what he supposed to be blood), which ended in slight vomiting. He now obtained a few minutes repose. Suddenly, however, his features contracted, his eyes opened with a hideous aspect, and, with maniacal force, he seized the hands of one of his attendants, whom his distempered imagination converted into an enemy—a taunting, bullying enemy. He wished to disembowel him, and ravingly talked of a deadly duel. In the imaginary contest, he believed himself to have received a large and mortal wound in the chest, into which as he supposed, he thrust his finger and enlarged the gory gash, that it might die the more quickly. Again he bade his friends adieu, and sank, oppressed, upon his bed. No voice was listened to or recognized.

Such was his condition during four anxious hours. Neither the sinapisms upon his feet, from which he experienced a sensation of prickling, nor the ice applied to his head, nor twenty leeches on either side of his neck, the bites of which bled profusely, could moderate his furious transports. They recommenced with additional violence.

Struck with the peculiar character of this delirium, which nothing could mitigate, and which contrasted strongely with the gentle disposition of the young man, I suspected that it must have originated in some violent *moral* influence. But upon this subject no one could give me any information. His delirious mind appeared to be wholly absorbed with the idea of a man—of an enemy—whose death he desired, even at the sacrifice of his own life. I thought of jealousy as the cause, and, in order to verify my suspicion, I placed my mouth to his ear, and said loudly, though not sufficiently so for others to hear, "*She prefers you: I am assured of it.*"

"Who told you so? Who are you to talk to me in this manner?" he instantly cried, with an expression of astonishment and fury. My conviction was from this moment established.

I learned that on the preceding day he had no inclination for dinner, was melancholy, and in the night had written a letter of ten pages. I inquired for this letter, and fortunately, the *commissionaire* who had carried it to the person addressed, was found. It as written to a young lady. Accompanied by the brother of the patient, I immediately went to her residence, and entreated her by every thing she held most dear, to ascertain if her presence in the chamber of the sick man would not exert a favorable influence, more powerful than any of the means theretofore resorted to. Through compassion and benevolence the lady overlooked those objections which she might have advanced against a compliance with my request. She went to the house of the young man, and when she entered his room he was as furious as he had been before.

"Why! Sir," said she, "what means all this?"

At these few words,—at the sound of that voice, a sudden change came over the features of my patient; a cloud as it were, fell from his eyes; his pupils, which had been large, contracted, and a smile softened the rigid outlines of his lips. He extended a hand on the side from which he heard the voice, and said, "Oh! is it you!" and as soon as he felt the hand of the lady placed within his own, he covered his eyes with the opposite hand and began to weep. I directed every one to leave the room, and went out myself.

I was absent but a few moments, and, upon returning, he extended his hand towards me and asked forgiveness. His reason had returned! His respiration was regular, and happiness beamed in his eyes. I interrogated him, in relation to the false impressions under which he had labored, but he could give no account of it. "All that I can remember," said he, "is, that my head became instantaneously relieved, the blood rushed towards my heart, and for a moment it was difficult to breathe. This oppression passed away with the few tears that I shed, and now I feel perfectly well."

He went out as usual on the following morning, and in the evening rode into the country. Since his return I have met him frequently, and he invariably expresses his gratitude for the almost miraculous manner in which he was restored.

*Am. Jour. of Insanity.*

